# Steaming soil is effective in eliminating invasive alien plants (IAPs) – part I: effect of exposure method

**DOI:** 10.1002/ps.8603

**Published:** 2024-12-12

**Authors:** Zahra Bitarafan, Wiktoria Kaczmarek‐Derda, Therese With Berge, Carl Emil Øyri, Inger Sundheim Fløistad

**Affiliations:** ^1^ Division of Biotechnology and Plant Health Norwegian Institute of Bioeconomy Research (NIBIO) Ås Norway; ^2^ Division of Forestry and Forest Resources Norwegian Institute of Bioeconomy Research (NIBIO) Ås Norway

**Keywords:** invasive species, resource recovery, thermal soil disinfection, weed management

## Abstract

**BACKGROUND:**

As regulations on pesticides become more stringent, it is likely that there will be interest in steam as an alternative approach for soil disinfestation. This study investigates the feasibility of utilizing a soil steaming device for thermal control of invasive plants.

**RESULTS:**

Seeds of *Echinochloa crus‐galli*, *Impatiens glandulifera*, *Solidago canadensis*, and rhizome fragments of *Reynoutria × bohemica* were examined for thermal sensitivity through two exposure methods: (1) steam treatment of propagative material in soil; (2) exposure of propagative material to warm soil just after heated by steam. Soil temperatures in the range of 60–99 °C and dwelling period of 3 min were tested. Increased soil temperature decreased seed germination/rhizome sprouting. The exposure method had a significant effect where higher temperatures were needed to reduce the seed germination/rhizome sprouting in method 2 explained by the effect of extra heat given in method 1. Using method 1, for *E. crus‐galli* and *S. canadensis*, the maximum mean temperature of approximately 80 °C was enough to achieve the effective weed control level (90%). This was lower for *I. glandulifera* and higher for *R. × bohemica*. Using method 2, 90% control was achieved at 95 °C for *S. canadensis*; more than 115 °C for *I. glandulifera*; and more than 130 °C for *E. crus‐galli* and *R. × bohemica*.

**CONCLUSION:**

Our findings showed a promising mortality rate for weeds propagative materials through soil steaming. However, the species showed varying responses to heat and therefore steam regulation should be based on the differences in weeds' susceptibility to heat. © 2024 The Author(s). *Pest Management Science* published by John Wiley & Sons Ltd on behalf of Society of Chemical Industry.

## Graphical abstract

As regulations on chemical pesticides become more stringent, it is likely that there will be interest in water steam as an alternative approach for soil disinfestation. This study investigated the feasibility of utilizing a soil steaming device for thermal control of selected invasive alien plants and showed a promising mortality rate for their propagative materials through soil steaming.

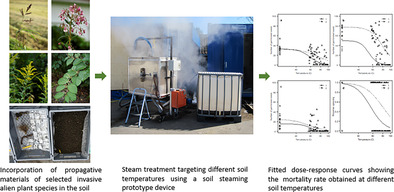

## 1 INTRODUCTION

Initially, soil disinfestation methods were developed with a general biocidal effect, such as the use of methyl bromide. The advantage of methyl bromide was its effectiveness in controlling a wide range of soilborne organisms, including nematodes, fungal pathogens, and many weeds.^1^ The removal of methyl bromide from the market, primarily due to its ozone‐depleting properties, has compelled the exploration, development, and re‐evaluation of alternative methods for soil disinfestation.^2^ The practice of soil disinfestation using steaming is undergoing reassessment in both open‐field and glasshouse horticulture due to its capacity for controlling soil‐borne pathogens, nematodes, and weed seeds. This method is believed to offer an additional advantage beyond pest control, as it avoids the negative environmental and worker health concerns associated with chemical fumigants.^3^, ^4^ The primary challenge, however, lies in determining the optimal dosage that effectively controls pests while minimizing any adverse impact on the soil's beneficial biotic and abiotic components. Furthermore, the adjustment needs to be tailored individually for each specific condition.^1^ With the previous evidence of soil steaming's effectiveness against pathogens and pests in both glasshouse and field conditions,^5^, ^6^, ^7^, ^8^, ^9^, ^10^ the methods may be a potential method for soil disinfection in the relocation and reuse processes of soil since the transportation and reuse of soils can introduce various plant diseases and pests to new regions and leading to their spread. In Norway, biologically contaminated soils are frequently disposed of in landfills and the rate of recycling for high‐quality purposes is relatively low. This is while enhancing resource efficiency and reducing climate impacts is essential for achieving global sustainable development. The reuse of soil has the potential to minimize the environmental consequences linked to sourcing new soil.^11^

Soil Steam International AS (Stokke, Norway) is a Norwegian company that is developing soil steaming machinery with the potential to recycle thousands of tons of soil directly on sites thus facilitating the safe reuse of soil that would otherwise require disposal due to pest spread. In this study, we present the outcomes of experiments aimed at evaluating the efficacy of steam treatment of soil against invasive plant species using a prototype of the soil steaming device with a method resembling the method in commercial products. These experiments are crucial in assessing the viability of scaling up the prototype to a potentially valuable tool for soil disinfection during soil relocation and reuse processes. Based on previous evidence of soil steaming's effectiveness against weeds in both glasshouse and field conditions as well as preliminary research using the same method,^12^, ^13^ we hypothesized that soil steaming will make a meaningful contribution to disinfecting soils from weeds/invasive alien plants (IAPs). The objective of this study was to determine the soil temperatures required to kill propagules (seeds and rhizome fragments) of selected IAPs when they are exposed directly or indirectly to heat during the steam treatment of the soil.

## 2 MATERIAL AND METHODS

In October 2021, a study was carried out in Ås, Norway to assess steam treatment to eliminate propagules of IAPs, either seeds or rhizomes, in soils.

### 2.1 Sample preparation before steaming

Mature seeds of *Echinochloa crus‐galli* (L.) P. Beauv, *Heracleum mantegazzianum* Sommier & Levier, *Heracleum persicum* Desf. ex Fisch., *Impatiens glandulifera* Royle, and *Solidago canadensis* L. were collected during September–October 2020 from Ås region (59.663° N, 10.790° E) in Viken county, southeast Norway. Seeds were collected from a high number of individual plants where possible. The pooled seeds were stored in paper bags under dry conditions at room temperature. For all treatments, four replicates of 100 seeds of each species were counted and placed into polypropylene fleece bags (9 cm × 7 cm). To break the dormancy, *I. glandulifera* seeds bags were chilled before the tests at 4 °C for 4 weeks in darkness. Seeds of *H. mantegazzianum* and *H. persicum* were covered with wet sand and chilled at 4 °C for 12 weeks in darkness. We also included the rhizome fragments of *Reynoutria × bohemica* Chrtek & Chrtková collected from the same region the day before the experiments in the study. Two rhizome lengths, 4–5 and 9–10 cm, were included in the experiments.

### 2.2 Steaming treatments

**Figure 1:**
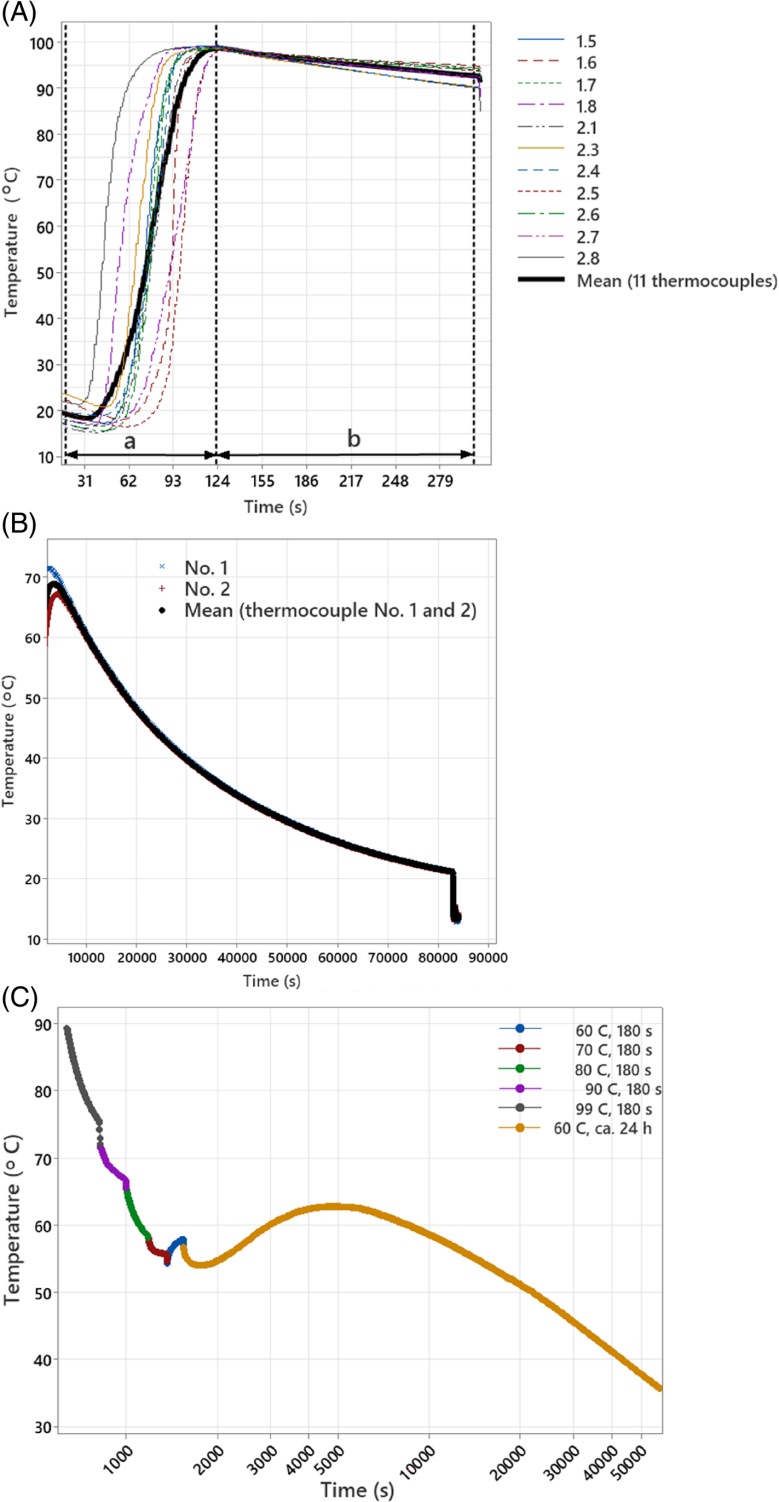
(A) Example of soil temperature curves for the target temperature of 90 °C using exposure method 1. Letters a and b show the periods with steam entering the steaming container and the post‐steaming dwelling period of 3 min, respectively. The average soil temperature was calculated from the measured temperatures by 11 of the thermocouples placed in the soil in each basket (one replicate) placed in the steaming container at the time. The maximum value of the mean soil temperature curve during the thermal treatment period (period a + period b) was extracted and used for further statistical regression analysis. By the end of the period b, the basket with the samples were removed from the steaming container. Samples were then removed immediately from the basket and the warm soil. For the approximately 24 h treatment using method 1 (B), however, by the end of period b, the basket was transferred from the steaming container to a closed Styrofoam box. The soil temperature was recorded by two thermocouples in the Styrofoam box. The maximum value of the mean soil temperature curve measured by the two thermocouples during the approximately 24 h resting time was then extracted and used for further statistical regression analysis. (C) Example of mean soil temperature curve based on two thermocouples for the various target temperatures using exposure method 2. Note that the *x*‐axis is transformed. Samples of the same replicate were exposed to warm soil in one Styrofoam box for 3 min each, except for the last which stayed about 24 h. The maximum value of the mean soil temperature curve for each target temperature was extracted and used for further statistical regression analysis.

To find the effective soil temperature to kill propagative material of selected species, we considered five target soil temperatures of 60, 70, 80, 90 and 99 °C and a dwelling period of 3 min. The dwelling period is indicated as b in Fig. 1(A). For the target temperature of 60 °C, two dwelling periods were included: 3 min and approximately 24 h. The temperature of 25 °C was considered for untreated control samples. Two exposure methods were considered. In exposure method 1, propagules were exposed to heat both during the steaming process itself (in which duration varied for each target temperature) and the following dwelling period of 3 min, that is, the total period with thermal treatment was more than 3 min. In exposure method 2, propagules were not present during the steaming but were only exposed to the warm soil after the steaming had stopped, that is, the total period with thermal treatment was 3 min. Four replications were used for each treatment. The experiment was conducted two times. The first run of the studies was on 20 October (exposure method 1) and 21 October (exposure method 2). The second run of the studies was conducted on 27 October (exposure method 1) and 28 October (exposure method 2). A commercial soil intended for use in public areas was used for the experiments (‘Lindum Anleggsjord’, Lindum AS, Drammen, Norway). It was composed of sand, composted sludge and garden waste, fertilized with mineral nitrogen and sulphur, with a pH of 8.2. It was characterized as a silty coarse sand soil (8% gravel, 70% sand, 14% silt, 5% clay, and 3% organic matter) with a high carbon/nitrogen (C:N) ratio (16) and a moisture content of 47% before being subjected to steaming.

In exposure method 1,^12^, ^13^ all bags including propagative material in the same replicate of a target temperature were placed at the bottom of a plastic perforated basket container (60 cm × 40 cm × 20 cm) and covered with a 7 cm soil layer. A total of 20 baskets (five target soil temperatures × four replicates) were used. Each one of the baskets was placed in the steaming container (190 cm × 144 cm × 88 cm, 130 kg, effective steaming area of 120 cm × 80 cm) at the time and 11 thermocouples were placed in its soil. When the container lid was closed, steam was released from the top with a constant temperature of approximately 150 °C and vacuumed from the bottom of the container. Soil temperature was monitored and recorded every second by means of PT1000 sensors connected to a cRIO‐9073 data logger (National Instruments, Austin, TX, USA). A custom‐made programme was used to record temperature, duration of the period with steam entering the steaming container, and the length of post‐steaming exposure duration. Steaming and vacuum were stopped when the average temperature was about 5–10 °C below the target soil temperature because the average temperature usually increased for some seconds after the steam was stopped. The basket was removed from the steaming container when the post‐steaming period of 3 min was completed. Samples were then removed immediately from the basket into ambient air temperature. For the target soil temperature of 60 °C followed by a resting period of approximately 24 h, the basket was removed from the steaming container after the completion of the post‐steaming dwelling period of 3 min and placed in a closed Styrofoam box (59.5 cm × 38.8 cm × 26.2 cm) for about 24 h. A total of four baskets (one target soil temperature × four replicates) which were smaller than the baskets in the other treatments were used. A separate Styrofoam box was used for each basket. Soil temperature was recorded by two thermocouples in each replication of the 24‐h treatment.

In exposure method 2, the soil was subjected to the same steaming procedure without the inclusion of propagative material in the baskets. This method was considered to mimic the thermal conditions in soil masses while propagative material are not in direct contact with the warm water steam itself. Soil was kept within two large mesh bags placed as two horizontal approximately 7 cm layers in the basket. When the average soil temperature reached the maximum possible (i.e., 99 °C), the basket was removed from the steaming container and immediately placed in a closed Styrofoam box to reduce the drop in soil temperature. Then a mesh bag including propagative material was immediately and rapidly inserted between the two layers of warm soil in the Styrofoam box and kept there for 3 min. When the temperature dropped to the next target temperature, another sample bag was inserted for 3 min, and this process continued until all bags in the same replicate of all target temperatures had been exposed to the warm soil. For the extra treatment of target soil temperatures of 60 °C with an exposure duration of 3 min following a resting time of approximately 24 h, the soil was left in the Styrofoam box after the completion of the post‐steaming exposure duration of 3 min. Soil temperature was recorded by two thermocouples in each replication.

### 2.3 Survival tests after steaming treatment

Bags including the propagative material were transported to the glasshouse after the steaming test. Each bag was opened carefully and placed on the soil surface in 12 cm diameter pots and covered by a thin layer (2–3 mm) of soil. Commercial potting soil was used (Tjerbo torvfabrikk AS, Rakkestad, Norway). It was composed of 80% sphagnum peat, 10% composted bark, 10% sand (*v/v/v*), limed and fertilized with NPK (950:40:220 mg L^−1^) with a pH of 5.5–6.5. Pots were placed randomly in a glasshouse [21 °C:16 °C and 14 h:10 h for day/night; relative humidity (RH) 68%] and watered from the bottom with tap water, and thereafter as needed during the experimental period. Seed germination/rhizome sprouting was followed for a month.

### 2.4 Post‐processing of soil temperature data

The recorded temperature data saved in csv format was imported and managed in Excel and Minitab.

Exposure method 1: The individual temperature measurement by each of the thermocouples was used to calculate the average soil temperature for each target soil temperature and replicate. An example of one replicate with a target temperature of 90 °C is shown in Fig. 1(A). The maximum values of each of the mean soil temperature curves during the thermal treatment period (steaming process plus the dwelling period) were extracted and used in the statistical analysis.

For the approximately 24 h treatments, the mean soil temperature was calculated from the two thermocouples per replicate. An example of one replicate is shown in Fig. 1(B). The maximum value of the mean soil temperature curve was higher than the maximum mean value reached during the 3‐min period inside the steaming container, except in one case where they were equal. The maximum value for each replicate was extracted and used in the statistical analysis.

Exposure method 2: Warming the soil by steaming it, temperature was followed in the same way as in method 1. During the exposure of propagules to warm soil in the Styrofoam box, the temperature was recorded by two thermocouples per replicate. Since the temperature data had time stamps, we could allocate the temperature data to the different bags. An example of one replicate of exposure method 2 is shown in Fig. 1(C). The maximum mean value during the 3 min periods for each sample was extracted and used in the statistical analysis.

It was generally difficult to reach the exact target temperatures. For the target temperatures of 60, 70, 80, 90 and 99 °C, the actual maximum mean soil temperatures were 56.6–82.8, 58.1–84.4, 61.1–88.7, 63.8–97.6 and 80.2–98.7, respectively. However, because the actual temperatures reached (56.6–98.7 °C) were in the same range as the target range (60–99 °C), the statistical regression analysis was considered sound. The length of the post‐steaming period of 3 min (180 s) was generally obtained except in one case of 221 s. However, this did not significantly affect the results. For the 24‐h treatment, the actual lengths of the prolonged dwelling periods were in the range of about 20.5–24.5 h.

### 2.5 Statistical analyses

For each species, the response to the soil temperature range in each method was described by a three‐parameter log‐logistic dose–response curve model^14^:

(1) $$y=c+\left(d–c\right)/\left(\left(1+\exp\;b\;\left[\log\;\left(x\right)-\log\;\left(\mathrm{ED}50\right)\right]\right)\right)$$

where *y* is the response and depends on the dose *x*. The temperature of 25 °C was considered for untreated control samples. The parameter *b* denotes the relative slope around the point of inflection, which is ED50, the dose required to reduce the response halfway. The parameter *c* denotes the lower limit of the curve (0). If the curve is decreasing from the upper limit *d*, *b* is positive, and if increasing toward *d*, *b* is negative.

We fitted a two‐parameter log‐logistic model for binomial data where the response was rhizome fragments sprouting (1 = upper limit) or not (0 = lower limit).^14^

The models were fitted using the extension package ‘drc’, for the software environment R.^15^, ^16^ The assessment of the individual fits was done by inspecting the graphical analysis of the residuals. *Post hoc* comparisons of parameters were based on pairwise *t*‐tests adjusted for multiple testing using the single‐step approach (Tukey's range test) implemented in the extension package drc multcomp.^17^

Since there was not a significant difference between the two rhizome lengths of *R. × bohemica*, data were pooled together for analysis. Due to the low germination in control samples of *H. mantegazzianum* and *H. persicum*, these two species were not considered for further analysis.

## 3 RESULTS

**Figure 2:**
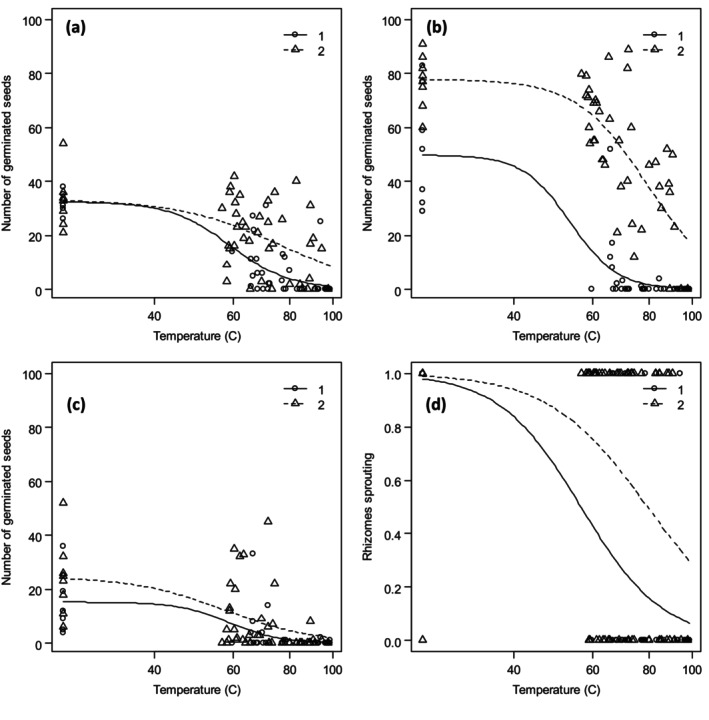
Fitted dose–response curves of Eqn (1) to the observed seed germination/rhizome sprouting (circles and triangles) of (a) *Echinochloa crus‐galli*, (b) *Impatiens glandulifera*, (c) *Solidago canadensis*, and (d) *Reynoutria × bohemica* as a function of the maximum soil temperature imposed by steaming soil through two different steaming methods, method 1: steam treatment of propagative material covered by soil and method 2: exposure of propagative material to warm soil following the steam treatment. The straight line and the dashed line show method 1 and method 2, respectively.

Generally, increased soil temperature following the steam treatment decreased seed germination/rhizome fragments sprouting in studied species (*P* = 0.001). Figure 2 shows the log‐logistic dose–response curves for tested weed species explaining the variation of the data within reasonable limits. Despite the low germination percentage for *S. canadensis* and *E. crus‐galli*, the same trend of decreasing seed germination by increasing temperature was achieved. The steaming method significantly affected the seed germination/rhizome sprouting of the selected weed species (significant at *P* = 0.001 except for the method effect for *S. canadensis* which was significant at 0.1) where higher temperatures were needed to reduce the seed germination/rhizome sprouting in method 2 compared to method 1 (Table 1) highlighting the effect of heat during the steam supplying on the plant propagative material death. Tables 1 and 2 show estimated effective doses for each of the steaming methods and the parameter estimates of the model, respectively. The slope *b* (steepness of the curve, Eqn (1)) is positive for all species as the curve decreases from the upper limit *d* and the response curve for steaming method 2 has a lower relative slope for all species (Table 2). Species responded differently to the soil temperature following the steam treatment (Fig. 1, Tables 1 and 2). In method 1, for the two species *E. crus‐galli* and *S. canadensis*, the soil temperature of approximately 80 °C lasting for 3 min was enough to achieve the effective weed control level (90%) (Table 1). This temperature was lower for *I. glandulifera* (approximately 70 °C) and higher for *R. × bohemica* (approximately 90 °C). In method 2, this temperature was 95 °C for *S. canadensis*; more than 115 °C for *I. glandulifera*; and more than 130 °C for *E. crus‐galli* and *R. × bohemica* (Table 1). In the approximately 24‐h treatment, no germination happened for *I. glandulifera*, *S. canadensis*, and *R. × bohemica*. However, few seeds germinated in the case of *E. crus‐galli*; in method 1, two seeds out of 800 tested seeds (in two different samples) and in method 2, 16 seeds out of 800 tested seeds (in one sample).

**Table 1:** Effective temperature (°C) required to reduce the weed seed germination/rhizome sprouting by 50% (ED50) and 90% (ED90) between the upper and lower limits of the log‐logistic dose–response curve model (Eqn (1))

Weed species	Exposure method	ED	Estimate (± standard error)	Lower limit	Upper limit
*Impatiens glandulifera*	1	50	53.4 (±11.08)	31.4	75.4
		90	69.8 (±5.8)	58.2	81.3
	2	50	79.2 (±3.1)	73.1	85.4
		90	117.3 (±9.5)	98.3	136.3
*Solidago canadensis*	1	50	58.6 (±13.8)	31.2	86.1
		90	78.3 (±13.4)	51.4	105.1
	2	50	57.008 (±8.5)	40.1	73.9
		90	95.2 (±17.5)	60.3	130.09
*Echinochloa crus‐galli*	1	50	58.9 (±6.9)	45.1	72.74
		90	82.9 (±9.1)	64.7	101.02
	2	50	75.7 (±6.6)	62.4	89.03
		90	133.8 (±29.6)	74.9	192.7
*Reynoutria × bohemica*	1	50	56.4 (±6.6)	43.2	69.6
		90	89.6 (±10.3)	69.2	109.9
	2	50	79.5 (±4.4)	70.6	88.3
		90	138.2 (±30.9)	77.1	199.2

Exposure methods 1 and 2 are steam treatment of propagative material in soil and exposure of propagative material to warm soil just after heated by steam, respectively.

**Table 2:** Estimated means of parameters of the log‐logistic function describing the relationship between seed germination/rhizome sprouting of the weeds and soil temperature (Eqn (1))

Weed species	Exposure method	Estimated parameter (± standard error)	
		*b*	*d*
*Impatiens glandulifera*	1	8.2 (±6.5) **	49.8 (±5.1) **
	2	5.6 (±1.1) **	77.8 (±4.9) **
*Solidago canadensis*	1	7.6 (±8.7)	15.2 (±3.4) **
	2	4.2 (±2.3)	24.6 (±4.2) **
*Echinochloa crus‐galli*	1	6.4 (±3.6)	32.4 (±3.5) **
	2	3.8 (±1.5) *	33.2 (±4.04) **
*Reynoutria × bohemica*	1	4.7 (±2.02) *	‐
	2	3.9 (±1.3) **	‐

Parameter *b* denotes the relative slope around ED50, and parameter *d* denotes the upper limit (the maximum seed germination/rhizome sprouting). Exposure methods 1 and 2 are steam treatment of propagative material in soil and exposure of propagative material to warm soil just after heated by steam, respectively.

Significance codes: ‘*’ ≤ 0.05 and ‘**’ ≤ 0.01.

## 4 DISCUSSION AND CONCLUSIONS

Numerous specialized machines have been designed with the purpose of utilizing steam for soil disinfection^4^, ^18^, ^19^, ^20^, ^21^ with positive results for agricultural and horticultural purposes.^5^, ^6^, ^7^, ^8^, ^9^, ^10^ The positive outcomes from these trials have encouraged further exploration and adoption of steam‐based soil disinfection as a promising and environmentally friendly technique for enhancing soil health. The research conducted by Miller *et al*. demonstrated that the act of physically mixing steam with soil was more effective in comparison to applying steam to still soil.^22^ These significant findings were later incorporated into the design and development of the steam applicator prototype constructed in 2011. Fennimore and Goodhue stated that soil steaming effectively managed soil pathogens and weeds while enhancing the vigour of plants grown in steamed soil right from the outset that steam had been used to control soil pests in the United States.^3^

Baker suggested that a temperature of 140 °F (±60 °C) maintained for a duration of 30 min in a steam treatment can potentially eliminate a wide range of plant pathogenic microorganisms, insects, viruses, and weed seeds present in the soil.^23^ In 2003, the efficacy of low temperatures was reported in a laboratory setting against weeds (*Chenopodium album* L. and *Agropyron repens* (L.) P. Beauv.) by Van Loenen *et al*.^8^ The lethal temperatures for these organisms in the imbibed state did not exceed 60 °C after an exposure time of 3 min followed by an 8‐min resting time in treated soil. In the dry state, however, they reported that the maximum temperature was necessary for achieving efficacy.^8^ In our study, we did not get a good control level at 60 °C in both methods when we had the dwelling period of 3 min but a good control level was achieved when we had the resting time of 24 h. The positive effect can be attributed to the resting time where we had 100% control of *I. glandulifera*, *S. canadensis*, and *R. × bohemica*. Though it was not 100% for *E. crus‐galli*, the number of germinated seeds was very few (for method 1 and method 2, 2 and 16 out of 800 tested seeds, respectively). The ability of barnyardgrass (*E. crus‐galli*) seeds to withstand high temperatures has traditionally been ascribed to factors such as the seed structure, protection of caryopsis by its palea and lemma, sterile floret, the second glume and partially by the first glume.^24^

Earlier studies with the same prototype showed soil steaming at temperatures ranging from 62 °C to 68 °C reduces seed germination of *E. crus‐galli* populations by 50%, while at higher temperatures of 76 °C to 86 °C, it effectively decreases germination by 90%.^13^ Soil steaming at a target temperature of 99 °C for durations of 90, 180, or 540 s resulted in no germination of weed seeds.^13^ This is also achieved in the current study for *E. crus‐galli* using the same method in the previous study (method 1) where supplying the steam provided extra heat to the seeds. De Oliveira *et al*. reported a soil temperature of 61 °C lasting for 5 or 20 min following the steam treatment did not significantly reduce Barnyardgrass and Palmer amaranth (*Amaranthus palmeri* S. Watson), but reduced Yellow nutsedge (*Cyperus esculentus* L.) and Large crabgrass (*Digitaria sanguinalis* (L.) Scop.) over 2 years in tomato fields; the treatment, however, led to the highest yield of fresh tomato weight per unit.^25^ Studies showed several species of rhizomes were also killed by exposing them to temperatures of approximately 60 °C for 5 or 10 min.^26^, ^27^

To define the lower limit of effectiveness we considered low target temperatures of 60 and 70 °C. Increasing temperature resulted in decreasing seed germination ability of *E. crus‐galli*, *I. glandulifera*, and *S. canadensis* as well as sprouting of rhizome fragments of *R. × bohemica* (Table 1 and Fig. 2). Seed germination of *I. glandulifera* and the sprouting of rhizome fragments of *R. × bohemica* were inhibited by temperatures above 64 °C and 79 °C, respectively, in earlier studies with the same prototype.^12^ The differences in the results of studies on the efficiency of steaming to control weeds can be attributed to different steaming methods, experimental conditions, and weed species. The temperature and exposure time are the most important factors that have been reported to affect the efficiency of steaming.^6^ While some research has shown that the primary factor in reducing heat exposure times is the maximum temperature required for killing,^24^, ^28^ an inverse relationship has been reported to exist between optimal temperature and exposure time.^29^, ^30^ Some other factors showed an effect in different studies are for example soil characteristics such as texture and humidity,^4^, ^6^, ^30^, ^31^ seed moisture content,^5^, ^6^, ^32^ seed structure, anatomy and morphology,^33^, ^34^ and high primary dormancy in propagules of weeds.^7^, ^32^ However, the relative influences of any individual affecting factor are difficult to detect,^31^ and maximum temperature and heat duration are considered foremost for germination reduction.^24^

In conclusion, utilizing steam for soil disinfection for relocating soil purposes can effectively hinder the introduction of propagative material of weeds/IAPs to new areas. Our findings showed a promising control level of invasive plant species through soil steaming. However, the species exhibited varying responses to temperature and duration and therefore steam regulation should be based on the differences in plant propagative materials' susceptibility to heat. While it has been suggested that steaming might not be the most cost‐effective method for this purpose, it is potentially a more sustainable approach in the long term.^25^ The potential negative impacts of steaming on microarthropods, microorganisms, and the natural soil microflora is also an important issue that should be considered, based on how the soil will be utilized in the future.^35^, ^36^ Steaming has been suggested to have minimal or no long‐term impact on soil quality or soil microbial communities.^36^, ^37^, ^38^ Certain studies, however, have documented a notable alteration in soil microbial activity resulting from steam disinfestation.^39^, ^40^ The variations observed could be attributed to factors such as the duration of steam application, the soil temperatures achieved during steam treatments, and the content of organic matter in the soil.^3^ In our study, we did not evaluate the impact of steaming on soil quality or soil microbial communities. Overall, the additional benefit of enhanced crop growth and yields has been however offered for steam treatment.^41^, ^42^ We did not also include energy consumption and cost calculations in our study. Additional research is considered to explore the impact of variables like soil type and moisture content on the mortality of weeds/IAPs propagative materials. Furthermore, it is crucial to examine whether steaming might yield any adverse consequences for the future utilization of soils.

## CONFLICT OF INTEREST STATEMENT

No conflict of interest has been declared by the authors. The funders had no role in the design of the research, data collection and analysis, writing of the manuscript, or the decision to publish the results.

## Data Availability

The data that support the findings of this study are available from the corresponding author upon reasonable request.
